# Role of Annexin 7 (ANXA7) as a Tumor Suppressor and a Regulator of Drug Resistance in Thyroid Cancer

**DOI:** 10.3390/ijms252313217

**Published:** 2024-12-09

**Authors:** Alakesh Bera, Surya Radhakrishnan, Narayanan Puthillathu, Madhan Subramanian, Nahbuma Gana, Eric Russ, Harvey B. Pollard, Meera Srivastava

**Affiliations:** 1 Department of Anatomy, Physiology and Genetics, Uniformed Services University of the Health Sciences, Bethesda, MD 20814, USAnahbuma08@gmail.com (N.G.); eric.russ@usuhs.edu (E.R.); harvey.pollard@usuhs.edu (H.B.P.); 2 Department of Anesthesiology, Uniformed Services University of the Health Sciences, Bethesda, MD 20814, USA

**Keywords:** ANXA7, p21, BRAF E600V mutation, p53 mutation, chemoresistance, Nutlin-3A

## Abstract

Thyroid cancer is the most common endocrine malignancy in the United States, with an overall favorable prognosis. However, some patients experience poor outcomes due to the development of resistance to conventional therapies. Genetic alterations, including mutations in BRAF, Met, and p53, play critical roles in thyroid cancer progression, with the BRAF V600E mutation detected in over 60% of cases. This study investigates the tumor-suppressive role of Annexin A7 (ANXA7) in thyroid cancer, focusing on its potential impact on tumor behavior and therapeutic response. Our analysis, which included RNA sequencing and protein profiling, revealed reduced ANXA7 expression in thyroid cancer cells, particularly in those harboring the BRAF V600E mutation. Upon treatment with inhibitors targeting BRAF and MEK, ANXA7 expression increased, leading to reduced phosphorylation of ERK and activation of apoptotic pathways. Additionally, we identified the cyclin-dependent kinase inhibitor p21 as a key player in modulating resistance to BRAF inhibitors. Combination therapies aimed at concurrently increasing p21 and ANXA7 levels resulted in a marked enhancement of apoptosis. These findings suggest a previously uncharacterized regulatory network involving the ANXA7/p21/BRAF/MAPK/p53 axis, which may contribute to drug resistance in thyroid cancer. This study provides new insights into overcoming resistance to BRAF and MAPK inhibitors, with implications for treating thyroid cancer and potentially other BRAF-mutant tumors. Future efforts will focus on high-throughput screening approaches to explore ANXA7-targeted therapeutic strategies for thyroid cancer.

## 1. Introduction

Thyroid cancer (TC) is one of the most common malignancies in the United States. This disease also has one of the highest survival rates with 98.2% of people surviving in 5 years [1]. Although most TC can be treated successfully with surgery, radioiodine ablation, or thyroid suppression therapy, tumor recurrence occurs in 5% to 20% of patients [2]. If distant tumor recurrences occur, the 10-year survival rate of TC is reduced to approximately 40%. The development of molecular determinants of disease recurrence has the potential to improve the clinical management of patients with TC by assisting in risk stratification and combined uses of more aggressive treatments with patients who are at higher risk of recurrence [3,4,5,6].

The BRAF V600E mutation is the most common genetic mutation detected in patients with TC [7]. A large multicenter retrospective study by Xing et al. found that, in an unadjusted analysis, the presence of the BRAF V600E mutation was significantly associated with TC-related mortality [8]. Another study by Kong et al. clearly showed the importance of the BRAF V600E mutation in making the TC drug-dependent [9]. The evolution of chemoresistance in patients undergoing treatment is a risk factor for more aggressive forms of the disease or worse outcomes [9].

The alteration of drug metabolism, derangement of intracellular pathways’ signaling, cross-talk between different membrane receptors, and modification of apoptotic signaling and interference with cell replication are all mechanisms that the cell uses to overcome the effect of pharmacological compounds [10]. Additionally, the activation of tyrosine kinase receptor systems’ specific signaling cascades leading to the activation of key pro-survival pathways such as PI3K/Akt/Ras/Raf/MEK/Erk, and STAT are implicated [11]. BRAF is a serine–threonine protein kinase, which plays a critical role in aggressive melanomas [12]. But the role of BRAF in less aggressive cancers, such as TC, is less known. We focused on the most well-known mutation BRAF V600E associated with drug resistance in this study on a TC model. BRAF V600E and aggressive clinic-pathological behaviors are highly correlated, as described by Xing et al. [8,13]. Thus, BRAF V600E does have a significant association with mortality, which likely occurs through promoting aggressive tumor behaviors. However, the BRAF mutation alone is not the key factor for the aggressiveness or chemoresistance of thyroid cancer. It is important to mention that p53, another crucial tumor suppressor gene, is also significantly mutated in thyroid cancer.

The tumor suppressor function of the calcium/phospholipid-binding Annexin-A7 (ANXA7) has been shown in ANXA7-deficient mice and validated in human cancers [14]. In the androgen-resistant prostate cancer cells, ANXA7 and p53 showed similar cytotoxicity levels. However, in the androgen-sensitive LNCaP, ANXA7’s level greatly exceeded the p53-induced cytotoxicity [14]. It is also important to mention that the ANXA7-GTPase is a tumor suppressor, frequently inactivated by genomic alterations at 10q21 [15]. In recent years, considerable amounts of data have accumulated describing the inactivation of ANXA7-GTPase in a variety of human malignancies and demonstrating the tumor suppressor potential of ANXA7-GTPase [16]. ANXA7-GTPase contains a calcium-binding domain that classifies it as a member of the annexin family [15]. The cancer-specific expression of ANXA7-GTPase, coupled with its importance in regulating cell death, cell motility, and invasion, makes it a useful diagnostic marker of cancer and a potential target for cancer treatment.

In this study, we investigated the relationship between BRAF V600E mutation, ANXA7 expression, and their roles in thyroid cancer progression and chemoresistance. We focused on thyroid cancer cell lines with the BRAF V600E mutation, which is known to contribute to drug resistance in various cancers, including thyroid and melanoma. We also identified a correlation between ANXA7 and p21, a cyclin-dependent kinase inhibitor involved in BRAF mutation-mediated ERK/MEK signaling.

The combination of BRAF and p21 inhibitors demonstrated a synergistic effect in overcoming resistance in BRAF V600E thyroid cancer cells, highlighting the potential for a novel therapeutic approach. This combination increased ANXA7 expression and enhanced apoptotic signaling, suggesting a tumor-suppressive ANXA7-BRAF-p21 axis. These results have translational significance, as early combination therapy targeting BRAF and p21 may improve outcomes in high-risk thyroid cancer patients and potentially extend to melanoma treatment. Understanding these resistance mechanisms can inform more effective therapeutic strategies for cancers driven by BRAF mutations.

## 2. Results

### 2.1. Expression of BRAF and ANXA7 in Different Tissues Including Thyroid Glands

**Figure 1:**
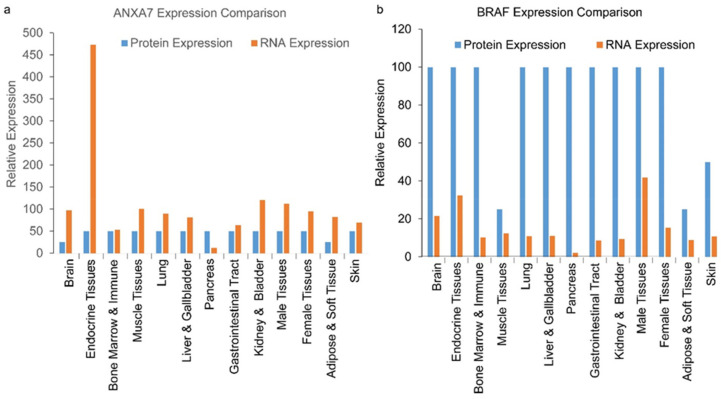

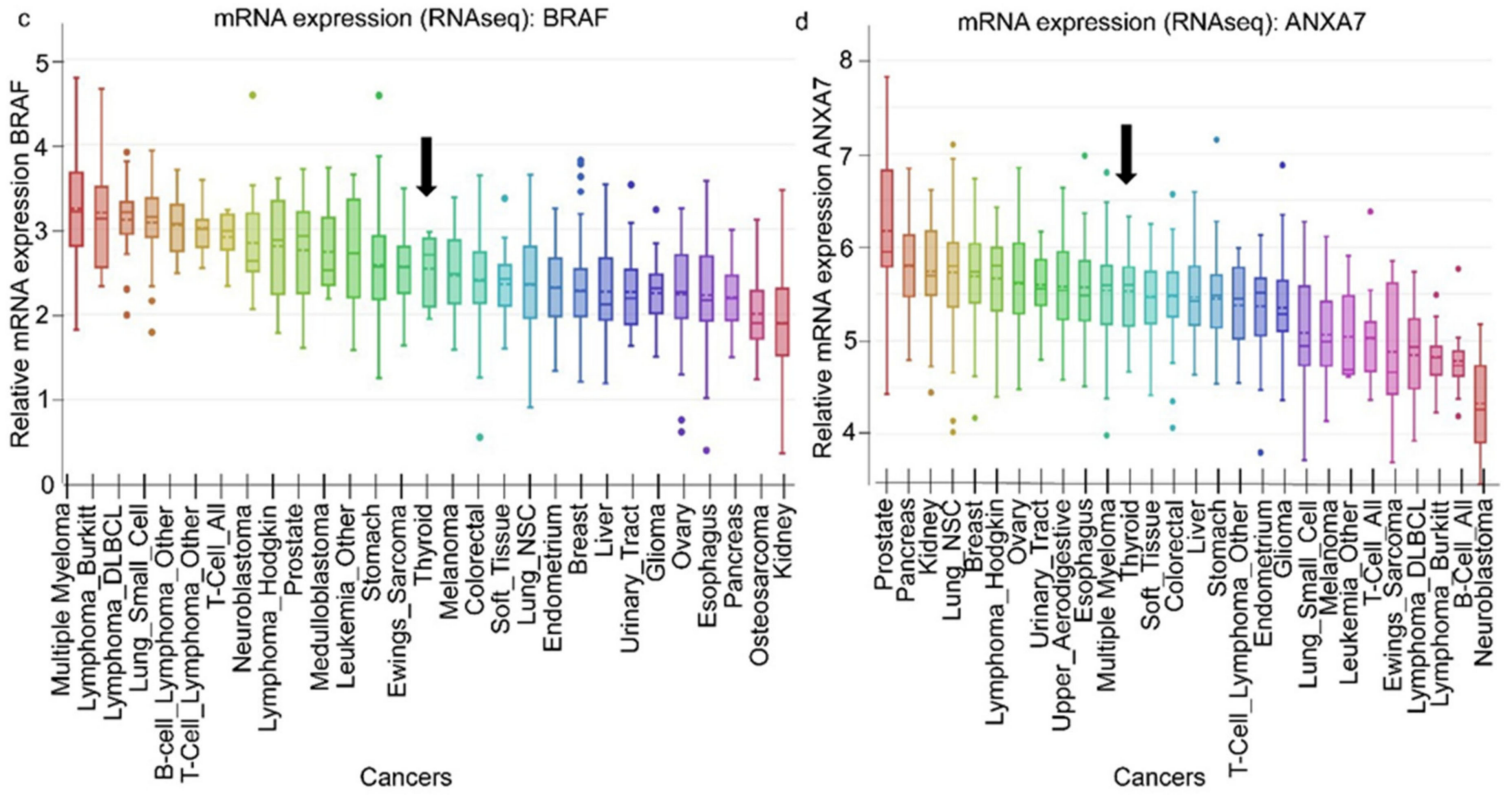
ANXA7 and BRAF expression levels. The protein and RNA expression of ANXA7 (**a**) and BRAF (**b**) were measured in various tissue samples and graphed. Panel a and b represent the expression of ANXA7 and BRAF in normal tissue. ANXA7 expression was dramatically higher in endocrine tissues (particularly in thyroid and parathyroid glands), relating to our discussion of thyroid cancer, and BRAF also had relatively high RNA expression in the endocrine (thyroid and parathyroid glands) tissues. When mRNA expression was determined in various cancers, the mRNA expression in thyroid cancer for BRAF (**c**) was mid-range in comparison to other cancers. This was similarly shown with ANXA7 (**d**), but ANXA7 mRNA expression was slightly higher than BRAF in thyroid cancer. Data analyses were performed with the publicly available dataset from the Human Protein Atlas.

**Figure 2:**
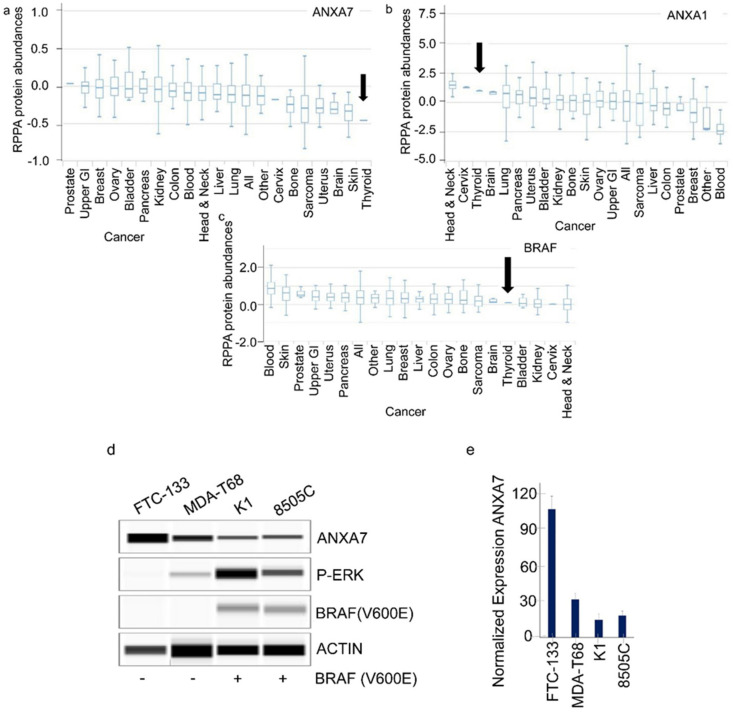
Protein expression in thyroid cancer and thyroid cancer cell lines. In the reverse-phase protein array (RPPA), the relative protein abundances detected in various cancers, ANXA7 expression (**a**) was lowest in thyroid cancer, though a related protein in the annexin family, ANXA1, had relatively high protein expression (**b**) in thyroid cancer. B-Raf (BRAF) protein expression (**c**) was relatively low in thyroid cancer. (**d**,**e**) In our own Simple Western analyses with the Wes instrument (ProteinSimple), four thyroid cancer cell lines were tested with relevant antibodies (**d**) to observe the relationship between ANXA7 and phosphor-ERK (P-ERK) in cells with and without the BRAF mutation. The ANXA7 expression levels in the four cell lines (**d**) were calculated from the area of the peaks at the expected molecular weights of the protein on the Compass software. It was normalized with the housekeeping protein to see higher expression in FTC-133 and MDA-T68 and lower expression in K1 and 8505C that have the BRAF V600E mutation.

In this current study, we analyzed the expression of BRAF and ANXA7 in different tissues, including the thyroid glands. We assessed the organ-wide expression of ANXA7 and BRAF RNA, as well as protein expression levels, by analyzing data from the Human Protein Atlas (www.proteinatlas.org) [17]. BRAF emerged as an important cellular protein with a significant expression level in almost all kinds of tissues, while the highest expression of ANXA7 in normal tissue was observed in the endocrine glands (thyroid gland, particularly in the parathyroid gland) (Figure 1a,b). This finding may explain the biological function of Annexin A7 (ANXA7), which belongs to the annexin family of calcium-dependent phospholipid-binding proteins. Additionally, we analyzed mRNA data from tumor samples for these two markers (BRAF and ANXA7). The data indicated that the expression of ANXA7 is lower in cancer samples, whereas the expression level of BRAF remains constant (Figure 1c,d and Figure S1). We also analyzed cell line data from the Cancer Cell Line Encyclopedia (CCLE) and from MCLP for these two proteins (Figure 2 and Figure S1). The protein expression data from cancer cell lines is consistent with the data from tumor samples. Specifically, the expression of ANXA7 is lowest within thyroid cancer cell lines compared to 19 other different cancer types, totaling over four hundred cancer cell lines (data analyzed from MD Anderson Cell Lines Project, https://tcpaportal.org/mclp/#/) [18].

To validate our findings, we performed wet laboratory experiments. We utilized four cell lines named FTC-133, MDA-T68, K1, and 8505C. Both K1 and 8505C have a BRAF V600E mutation, which was also verified with Western blot data using a specific antibody. The mutational status of *KRAS, TP53, BRAF, PIK3CA,* and *PTEN* in these cell lines and in other thyroid cancer cell lines are also mentioned in the Supplementary Data Table S1. The expression of ANXA7 in FTC-133 is very high, and the phospho-ERK expression is the lowest, while the inverse is true with the cell lines harboring the BRAF V600E mutation (Figure 2d,e). These results show that the inflammatory pathway is activated by the BRAF V600E mutation, which is confirmed by the expression of P-ERK in these two cell lines (Figure 2d,e).

### 2.2. The Regulation of ANXA7 Expression by BRAF V600E Mutation

**Figure 3:**
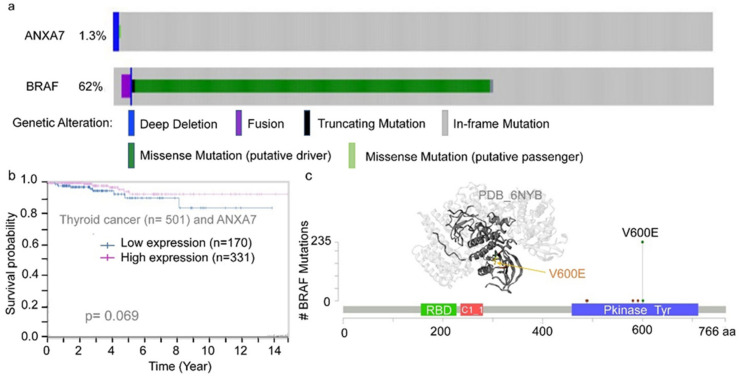
(**a**,**c**) ANXA7 and BRAF V600E mutation and alteration effects on cancer survival rate with respect to ANXA7 expression. For the BRAF mutation, the most common alteration is the missense mutation (V600E). In thyroid cancer patients with this alteration, patients had lower chances of being disease-free (Figure S1). Data analyses were conducted from TCGA dataset at the Human Protein Atlas portal, n = 501 and the structural features are also shown (**c**). (**b**) Thyroid cancer survival curve with respect to ANXA7 expression. Lower expression is associated with a poor survival.

To further investigate the regulatory role of the BRAF mutation (particularly V600E) on ANXA7 expression, we analyzed large-volume datasets from the curated databases cBioportal and CCLE. In about 60% of the thyroid cancer cases, BRAF mutations are present (Figure 3 and Figure S1 and Table S1 for cell lines). The BRAF gene is a proto-oncogene. The highest level of BRAF mutation is through the V600E mutation (cBioPortal). When the mRNA expression levels were viewed, both BRAF and ANXA7 had mid-range levels in thyroid cancer cases (Cancer Cell Line Encyclopedia, CCLE). However, when looking at the protein expression levels of these genes through reverse-phase protein array (RPPA), ANXA7 expression was lowest in thyroid cancer (MD Anderson Cell Line Project, Figure 2). Since it is the BRAF mutation and not the expression that leads to cancer, we decided to look more closely at ANXA7.

### 2.3. P-ERK and MEK Inhibitors Induce ANXA7 Expression by Activating the Apoptotic Pathway

The four cell lines, with MDA-T68, K1, 8505C, and FTC-133, were tested with antibodies with Simple Western analyses with the Wes machine. As indicated in Figure 2, these cell lines were shown to have varying levels of expression with the ANXA7, P-ERK, and BRAF antibodies. They were tested with BRAF V600E to confirm that K1 and 8505C were the two with the expression of the BRAFV600E mutation. These two also had higher P-ERK levels of expression, in comparison to MDA-T68 that had low expression of P-ERK and FTC-133 that had no noticeable expression. When ANXA7 expression was normalized among the four cell lines, FTC-133 had the highest expression, followed by MDA-768, whereas K1 and 8505C had the lowest levels of ANXA7 expression (Figure 2d,e). The inverse relationship between higher levels of BRAF V600E and ANXA7 can be seen when comparing these cell lines, indicating a tumor suppressive role in thyroid cancer that is inhibited with the aggressive and oncogenic BRAF V600E mutation.

**Figure 4:**
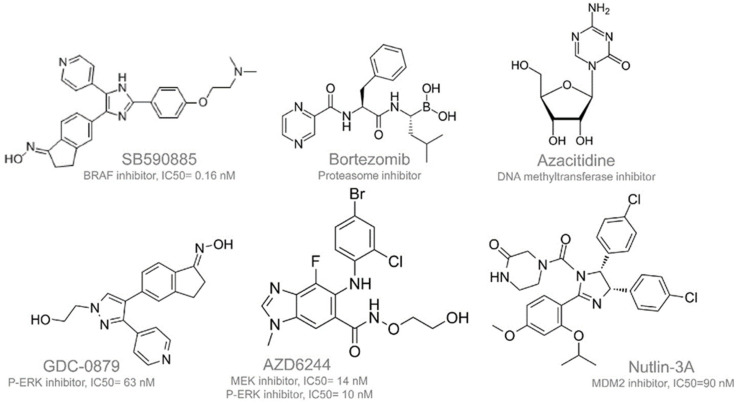
Structure of drugs tested. SB590885 inhibits BRAF; GDC-0879 inhibits P-ERK; and AZD6244 inhibits MEK and ERK1/2 phosphorylation. As per MCLP drug–protein relationship, Nutlin induces p21 and induces differentiation (Bortezomib is an FDA-approved cancer drug, proteosome inhibitor. These molecules were tested in equipotent concentrations to compare the relative biological activities.

**Figure 5:**
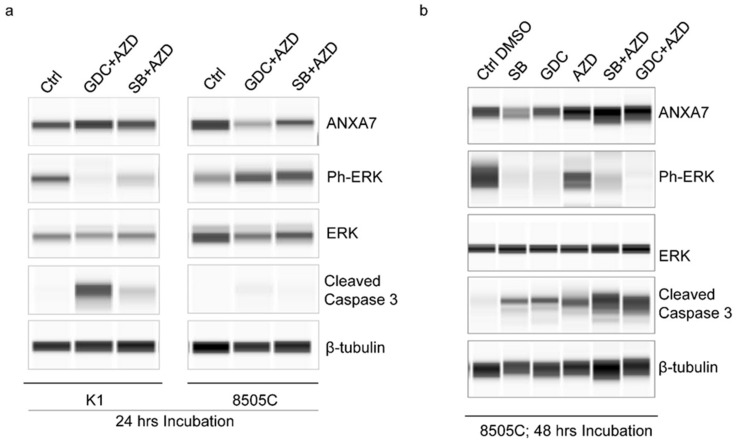
Time-dependent expression correlation of ANXA7. The ANXA7 expression is inversely correlated to Ph-ERK-induced oncogenic drive. Increased level of ANXA7 induced greater apoptosis of cancer cells. These levels were achieved during combination therapy and the highest levels were observed when incubated in combination drugs. (**a**) For both cell-lines K1 and 8505C at 24 h post incubation with drugs and, (**b**) Data with 8505C cell-line at 48 h post-incubation with drugs. Abbreviation: AZD = AZD6244 (1 µM); GDC = GDC0879 (0.1 µM); SB = SB590885 (1 µM).

Next, we pursued the experiments to inhibit P-ERK by different inhibitors. Since the BRAF V600E mutation plays a significant role in regulating the MAP kinase/ERK’s signaling pathway, which affects cell division, differentiation, and secretion [19], we used different inhibitors to block P-ERK activity. Specifically, the drugs SB590885, AZD6244, GDC-0879 (the list of drugs and their structures are in Figure 4 and Table 1) were used to test their effects on ANXA7 and P-ERK over a 24 h and 48 h period. These Simple Western assays were performed on both the K1 and 8505C cell lines that had the BRAF V600E mutation. As seen in Figure 5, the cell lines were treated with each drug alone and in combination and tested with ANXA7, P-ERK, ERK, and Cleaved Caspase 3 antibodies against a control. As primarily seen in 8505C, as K1, P-ERK increased and ANXA7 decreased when compared to the control in the first 24 h period. After the 48 h period, the P-ERK decreased with an increased ANXA7 expression with the drugs and mostly the drug combinations. This shift correlated with an increase in cleaved Caspase 3 (apoptotic marker) along with an increasing expression of ANXA7, as more cells were dying in the 48 h period. It is important to mention the potential for the development of resistance in cancer cells (8505C) when treated with inhibitors of the BRAF or MAPK pathways. These pathways play critical roles in cell growth, proliferation, and survival, and their dysregulation is often associated with cancer development and progression. However, cancer cells can develop mechanisms to bypass or counteract the effects of targeted therapies, leading to treatment resistance. The presence of surviving cells following drug treatment suggests the potential emergence of a resistant population within the cell lines.

### 2.4. The Increase in ANXA7 Expression Can Induce Apoptosis in Thyroid Cancer Cells

**Figure 6:**
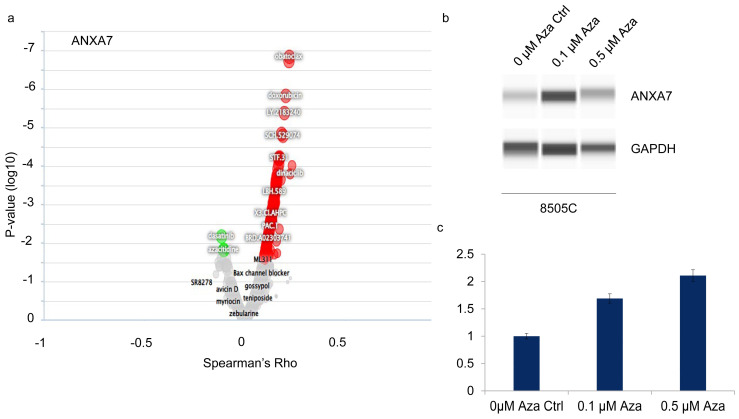
Expression of ANXA7 induced by drug. (**a**) From the correlation of drug–protein (data analysis from MCLP portal) we found Azacitidine (Aza) as the ANXA7-inducing drug. (**b**,**c**) We validated the expression of ANXA7 by incubating 8505C cells with Aza at different concentrations. Data indicated that increase in ANXA7 by implication of Aza is dose-dependent. Aza = Azacitidine.

As previously mentioned, ANXA7 expression is particularly important in the thyroid gland, where it is found at its highest levels. The biological functions of ANXA7 are likely linked to regulating thyroid gland activity, including maintaining calcium ion channels, facilitating hormone transport, and supporting various other cellular processes. Therefore, the decrease in ANXA7 expression may play a critical role in the development and progression of thyroid cancer. Both data from cell lines and patients’ samples indicated that ANXA7 decreases during pathogenesis. It is also hypothesized that the downregulation of ANXA7 expression could serve as an early and stage-specific biomarker for thyroid cancer or other thyroid-related malignancies [20]. Therefore, it is of paramount importance to identify a drug that could increase ANXA7 expression regardless of the mutation level of other corresponding genes. We analyzed data from the MD Anderson Cell Lines Project (MCLP) for the protein–drug relationship (Volcano graph, Figure 6a). This analysis indicated that the drug Azacitidine (in the figures indicated as “Aza”) upregulates ANXA7 and sensitizes the cells to apoptosis (Figure 4 for the structure of the drug and Figure 6 for its effect of drug on 8505C). The increase in ANXA7 in 8505C cells is observed in a dose-dependent manner with Azacitidine (Figure 6b). These results suggest that the upregulation of ANXA7 expression has the potential to trigger apoptosis in thyroid cancer cells.

### 2.5. Induction of p21 Through Nutlin-3A Drug Treatment

Understanding the mechanisms driving drug resistance is of paramount importance for enhancing cancer treatment outcomes. Combination therapies, which target multiple pathways concurrently or integrate immunotherapy, are being explored to combat or delay resistance development and enhance treatment effectiveness. In our investigation, we aimed to identify additional pathways that could serve as potential targets for combination therapy to overcome resistance induced by inhibitors of the BRAF/MAPK axis.

**Figure 7:**
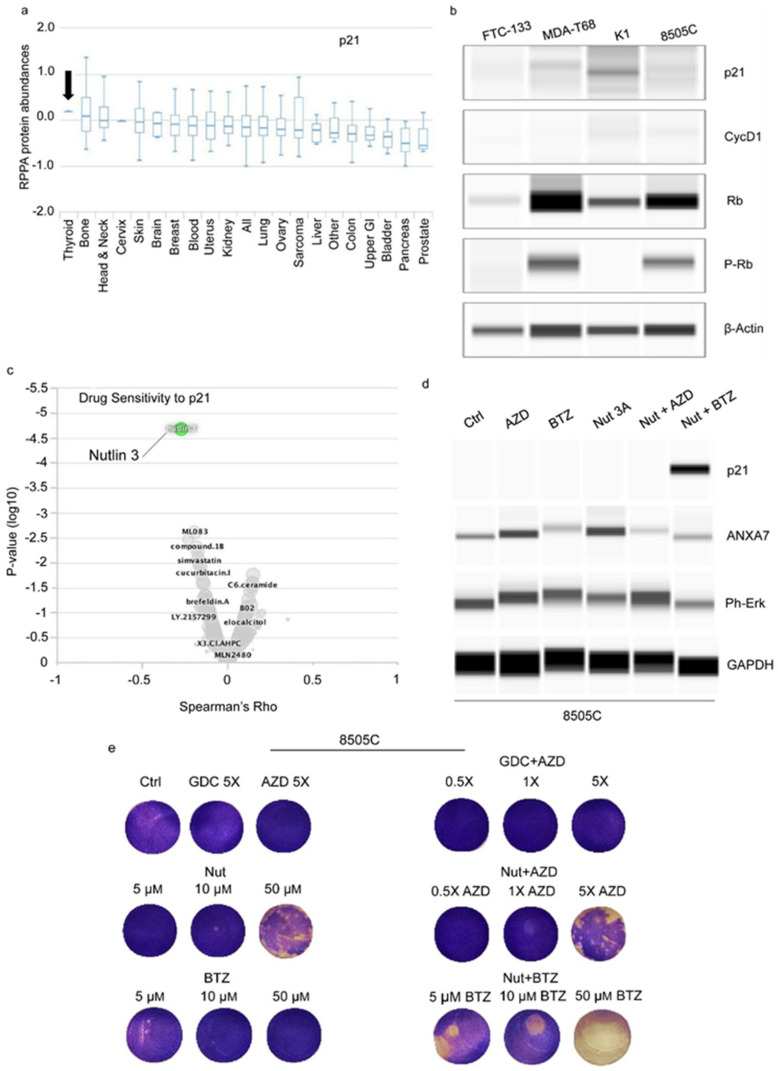
Involvement of BRAF/ANXA7 axis with p21 and combination therapy. (**a**) The protein expression level of p21 in different cancer cell lines. Data show the lowest expression in prostate cancer cell lines. (**b**) Expression of RB/p21-related protein expression level in four different thyroid cancer cell lines. (**c**) The p21 and drug relationship. Data indicating that the Nutlin will increase the expression of p21 and sensitize the apoptosis of the cancer cells. (**d**) A series of drugs response to 8505C cell apoptosis at 24 h incubation. (**e**) Crystal violet staining of the 8505C cells after incubating with different drugs for 24 h. The cells are resistant to GDC and AZD treatment alone. Cells are mildly sensitive to Bortezomib (BIZ) treatment in a dose-dependent manner. The Nutlin (Nut) inhibits growth and differentiates the cells. The Nut-only panel shows the more differentiated morphology by absorption of dendritic processes and contact inhibition. GDC + AZD treatment has some additive effect which increases the cell death but is not enough to completely overcome the resistance and complete apoptosis. Nut + AZD treatment is more potent—it induces differentiation and sensitizes the cells to chemotherapy. Nut + BIZ treatment completely overcomes the chemoresistance and all cells are killed and eradicated, as seen by the negative toward crystal violet staining. The panel shows no viable cells and only dense apoptotic bodies are visible. Cell ghosts are faintly visible in the background. Abbreviation: BIZ = Bortezomib; AZD = AZD6244 (1 µM, 1×); GDC = GDC0879 (0.1 µM; 1×); SB = SB590885 (1 µM; 1×).

Our previous study indicated that ANXA7 is linked to the RB/p21 axis and protects normal prostate cells and induces distinct patterns of RB-associated cytotoxicity in androgen-sensitive and -resistant prostate cancer cells [21]. In this study, we focused on p21, also known as cyclin-dependent kinase inhibitor 1 (CDKN1A), a pivotal protein regulating the cell cycle. Acting as a tumor suppressor, p21 inhibits cyclin-dependent kinases (CDKs), pivotal enzymes driving cell cycle progression [22,23,24]. Our study revealed that p21 expression levels were notably elevated in thyroid cancer cells, with distinctions observed based on the presence of the various BRAF V600E mutations (Figure 7a,b). Further analysis utilizing MCLP data elucidated the relationship between drugs and proteins, showcasing Nutlin’s ability to increase p21 expression and sensitize cancer cells to apoptosis (Figure 7c). This observation aligns with the existing literature demonstrating Nutlin’s capability to induce p21 across various systems [25,26,27]. We are utilizing Nutlin 3A over Nutlin 3 primarily due to its superior potency and efficacy. Nutlin 3A is an enantiomeric isoform that has been demonstrated to be highly potent in inhibiting the interaction between p53 and MDM2. This interaction inhibition is crucial for restoring p53 activity, which is a key tumor-suppressor mechanism.

Employing Nutlin 3A to upregulate p21, our results demonstrate successful modulation of p21 expression within our experimental systems. Notably, we uncovered a synergistic effect of Nutlin 3A when combined with P-ERK inhibitors (particularly Bortezomib), leading to augmented tumor cell death and complete suppression of colonies previously resistant to P-ERK inhibitors alone (Figure 7d,e). This novel discovery highlights Nutlin 3A as a potential pharmacological target for resistant thyroid cancers with poorer prognoses. Co-treatment with Nutlin 3A not only normalized ERK expression but also restored retinoblastoma (Rb) tumor suppressor activity, redirecting cellular processes toward differentiation and away from tumorigenesis.

In summary, our findings unveil p21 modulation, particularly through Nutlin 3A co-treatment, as a promising avenue for overcoming resistance in thyroid cancers, offering insights into novel therapeutic strategies to improve patient outcomes.

## 3. Discussion

The role of Annexin A7 (ANXA7) in thyroid cancer presents a complex but intriguing area of investigation, given its high expression levels in the normal thyroid gland and significant downregulation in thyroid cancer tissues (Figure 1 and Figure S1). ANXA7 is involved in several crucial biological functions within the thyroid, such as maintaining calcium ion channel activity, hormone transport, and regulating various cellular processes [15]. Our previous tissue microarray studies demonstrated that ANXA7 expression is notably lower in thyroid cancer tissues compared to other cancer types, suggesting a potential tumor suppressive function of ANXA7 in the thyroid. In this study, we explored the molecular mechanisms underlying the reduction in ANXA7 expression in thyroid cancer and its association with drug resistance pathways. Our findings suggest that the decreased expression of ANXA7 may contribute to the development and progression of thyroid cancer by impairing cellular mechanisms that normally inhibit tumor growth and metastasis. This reduction in ANXA7 may influence the survival of thyroid cancer cells, particularly under therapeutic stress, where drug resistance is a significant challenge.

The BRAF gene is also referred to as proto-oncogene and the protein is more formally known as serine/threonine-protein kinase [9,28]. This protein plays a role in regulating the MAP kinase/ERK’s signaling pathway, which affects cell division and differentiation [19]. Diseases associated with BRAF mutation include melanoma, cardio-faciocutaneous syndrome, lung cancer, and many other malignancies [19,29,30]. Evidence is also suggested that mutation of this protein is associated with over 60% of thyroid cancers [7]. B-type Raf (BRAF) kinase (V600E) mutation and p53 protein expression were evaluated in papillary thyroid cancer patients [31]. Thyroid cancer is the most common endocrine malignancy, and most patients with thyroid cancer have a great chance for success with treatment. There is, however, a group of patients with poor prognoses. The current data present the well-known cellular marker of normal thyroid and parathyroid gland and correlation with thyroid cancer. Further studies on new cellular thyroid markers are essential for understanding and improving diagnostic and prognostic capabilities in thyroid cancer.

Based on our clinical findings, we decided to look at the drug sensitivity patterns in cell lines with similarly known mutations. The emergence of drug resistance and potential addiction in cancer treatment, particularly associated with MAPK/ERK pathway inhibitors like MEK or BRAF inhibitors, presents a significant challenge in oncology. The complexity is further compounded by the presence of multiple cancer-related mutations such as KRAS or p53 alongside the BRAF (V600E) mutation [9,32,33,34].

Developing strategies to overcome resistance and enhance sensitivity to these inhibitors is indeed crucial. One approach involves combination therapies targeting multiple pathways or mutations simultaneously. For instance, a combination of MEK or BRAF inhibitors with drugs targeting KRAS or p53 pathways might help address resistance mechanisms and improve treatment outcomes. Our focus was to identify novel connections for the improvement in BRAF gene mutation-based therapy. Our results show that ANXA7, a known tumor suppressor gene that has been extensively studied by our laboratory for the past decade, is correlated to favorable outcomes. In this study, we observed that the upregulation of ANXA7 causes drug-induced chemo-sensitization to otherwise recalcitrant tumors with the BRAF V600E mutation. However, the exact mechanism which causes the tumor sensitization by ANXA7 expression remains to be clarified. The p21 induction has the same effect on the cell as the activation of p53, as it curtails the PI3-Akt-mediated activation of NF-kB, which is a well-characterized oncogenic driver in various cancers [35]. A previous study indicated that the bortezomib sensitivity increases by the combination of Nutlin 3 for the cancer cells having defective p53 expression [36]. We have shown that ANXA7 appears to be a rapamycin-like endogenous regulator that can balance cell growth and survival in homeostasis and tumorigenesis through mTOR/Akt/PI3K pathway components [16]. This pathway, along with the mTOR axis, is usually coactivated in BRAF mutations, which induces the vascular endothelial growth factor receptors (VEGFR) and platelet-derived growth factor receptors (PDGFR); these receptors are crucial in sustaining the tumor growth [37]. Overall, the ANXA7-mediated response is useful in the induction of pro-survival phenotypes, shedding light on potential therapeutic strategies for tumors associated with BRAF mutations.

In our subsequent analysis, we tested the correlation of p21 to the ANXA7 response. This might point to the conclusion that the ANXA7 response as a tumor suppressor might be acting through a different mechanism other than cell cycle arrest. Interestingly, the presence of the BRAF V600E mutation was directly correlated with ANXA7 expression. Thus, even though ANXA7 might not be directly related to p21, it might be acting through BRAF-mediated downregulation of the ERK pathway, which results in c-MYC normalization. It is well-characterized that c-MYC is a driver of cancer in its mutated forms [38]. Thus, drug therapy targeting p21 and ANXA7 upregulation will be a great tool in causing cell cycle arrest and increasing sensitivity of cancer cells to pharmacological agents. Our findings in this study suggest that the BRAF V600E mutation may be making the cells more invasive and more resistant by inhibiting the signaling of ANXA7. It is already known that the p53-mediated downregulation of p21 occurs in c-MYC overactivation [39]. ANXA7 upregulation may thus chemo-sensitize the tumor if p21 modulators are added to the treatment regimen, as they will counteract the c-MYC overexpression and oncogenic drive towards stemness, which has the same effect as c-MYC inhibition in these conditions. This finding may be generalizable to a large degree of cancers including melanoma and thyroid cancers where BRAF V600E is highly prevalent (Figures S1 and S2). The relatively simple characterization of the BRAF V600E mutation by PCR, and the expression levels by RT-PCR and Western analysis make these potential therapeutic strategies feasible for clinical translation.

The novelty in our current study lies in several key aspects related to cancer treatment related to the BRAF V600E mutation and particularly thyroid cancer and melanoma. Specifically, our study unveils a previously unidentified pathway implicated in thyroid malignancy and drug resistance, involving the ANXA7/p21/BRAF/p53/MAPK axis. This novel pathway sheds light on the intricate interplay between ANXA7 expression, BRAF/p53 mutation, and their impact on disease progression and aggressiveness, opening avenues for targeted therapeutic strategies.

Furthermore, our current research unveils insight into overcoming drug resistance. By investigating the interplay between ANXA7 expression and BRAF mutation (V600E), our study offers promising insights into overcoming resistance to the BRAF V600E mutation or MAPK-targeting drugs in the treatment of thyroid cancer. We demonstrate that treatment with the BRAF V600E mutation and MEK inhibitors leads to upregulated ANXA7 expression, decreased P-ERK levels, and increased apoptotic markers, indicating a potential strategy for overcoming drug resistance.

In addition, in this current study we discover a strategy for synergistic drug combinations to overcome the drug resistance. Our findings highlight the synergistic effects of combining drugs to elevate both p21 (Nutlin 3a) and ANXA7 levels, which enhances apoptotic signaling. Particularly, we observe a significant increase in bortezomib activity when combined with the p21 inducer Nutlin 3a molecule. This suggests a promising approach for enhancing the efficacy of existing therapies through targeted drug combinations.

Overall, our study offers novel insights into the molecular mechanisms underlying thyroid cancer progression and drug resistance, paving the way for the development of more effective therapeutic strategies for patients with BRAF-mutated thyroid cancer and potentially other malignancies.

## 4. Materials and Methods

### 4.1. Cells

Four thyroid cancer cell lines were chosen for the experiment—MDA-T68 acting as a wild type (lacking BRAF V600E mutation), K1 and 8505C having the BRAF V600E mutation, and FTC-133 cells having the PTEN deletion mutation. The cells were grown in DMEM/F12 (a 50:50 mixture of Dulbecco’s Modified Eagle Medium (DMEM) and Ham’s F-12 medium) with 10% serum (FBS) with the addition of penicillin and streptomycin to protect the culture from common contaminants. All cell lines were grown with the above media (DMEM/F12), except FTC-133, which was grown in RPMI-1640 supplemented with 10% FBS, as recommended by ATCC. Cultures were periodically tested for mycoplasma contamination, and sterility was ensured. The incubation was conducted at 37 °C with 5% CO_2_ in a humidity-maintained environment.

### 4.2. Capillary Electrophoresis and Immunoassay

Capillary electrophoresis and immunoassay or Simple Western analyses were performed using the Wes machine (ProteinSimple, Bio-Techne, San Jose, CA, USA) according to the manufacturer’s protocol [40,41]. Briefly, 1 µg total lysate samples was mixed with a master mix (ProteinSimple) to a final concentration of 1x sample buffer, 1× fluorescent molecular weight markers, and 40 mM dithiothreitol (DTT), and the samples were then heated at 80 °C for 5 min. The samples, blocking reagent, primary antibodies, HRP-conjugated secondary antibodies, chemiluminescent substrate, and separation and stacking matrices were dispensed to designated wells in a pre-designed plate (ProteinSimple). After plate loading, the separation electrophoresis and immune-detection steps took place in the capillary system and were fully automated. Simple Western analysis was carried out at room temperature and with the instrument on default settings with high dynamic range (HDR) for the chemiluminescent detection step. The details of antibodies and their corresponding dilutions are identified and listed in Table 2. The digital images were analyzed with Compass software (Compass version SW 6.3.0; Protein Simple), and the quantified data of the detected protein were reported in molecular weight.

### 4.3. Database, Bioinformatics Analysis, and Statistics

We performed mutational analysis using cBioportal [http://www.cbioportal.org] [42,43]. The results were reported in color-coded figures, which were based on an odds ratio of the presence of mutational frequency to the specific cancer type. The Cancer Cell Line Encyclopedia (CCLE—https://portals.broadinstitute.org/ccle) [44] database was used to study the transcriptomic relationship between BRAF and ANXA7. Relative protein abundance was calculated for BRAF and ANXA7 using the MD Anderson cell line project (MCLP, https://bioinformatics.mdanderson.org/main/MCLP:Overview, accessed on 6 August 2024) [18].

### 4.4. Reverse-Phase Protein Array (RPPA)

Reverse-phase protein array data were obtained from the publicly available MD Anderson Cancer Cell Line Project (MCLP) data portal [18]. RPPA is a high-throughput antibody-based technique used for quantitative measurement of protein expression and phosphorylation status across multiple samples. In this study, RPPA was utilized to assess the expression levels of specific proteins and phosphorylated proteins related to multiple cancer cell lines from various types of cancers. The following primary antibodies were used for protein detection: ANXA1: Mouse monoclonal antibody, BD Biosciences (Cat # 610066); ANXA7: Mouse monoclonal antibody, BD Biosciences (Cat # 610668); BRAF: Rabbit polyclonal antibody, Abcam (Cat # ab33899). Each antibody was validated for specificity and sensitivity according to the manufacturer’s instructions. Protein samples were spotted onto nitrocellulose-coated slides, and antibody binding was detected using appropriate secondary antibodies and signal amplification methods. Data normalization and quality control were performed as per the MCLP protocols to ensure accurate quantification of protein levels.

### 4.5. Crystal Violet Staining for Drug-Induced Killing of Thyroid Cancer Cells (8505C)

To assess drug-induced cell death, crystal violet staining was performed on 8505C thyroid cancer cells. Cells were seeded in 24-well plates at a density of 2 million cells per plate (approximately 83,000 cells per well) and left to adhere overnight in complete growth medium. The following day, the medium was replaced with 1 mL of serum-free Opti-MEM (ThermoFisher, Waltham, MA, USA; Catalog number 31985062) per well. Cells were then treated with the indicated concentrations of individual or combined drugs, as specified in the figures. After 24 h of drug treatment, the conditioned media were removed, and the cells were gently washed with phosphate-buffered saline (PBS). The cells were then fixed and stained with 0.01% crystal violet solution prepared by diluting a crystal violet stock solution (1 g of crystal violet powder (Sigma-Aldrich, Burlington, MA, USA; Cat # C6158) dissolved in 100 mL of methanol, then diluted 1:100 in distilled water). The crystal violet solution was added to each well, and the plates were incubated at room temperature for 30 min. Following staining, the wells were washed with distilled water to remove excess dye and left to air dry completely. Images of the stained plates were captured to document the results.

### 4.6. Drugs/Inhibitors

Most of the inhibitors were purchased from www.selleckchem.com and Nutlin-3A was purchased from Sigma-Aldrich. The drug concentrations and other details were as described in Table 1.

## Figures and Tables

**Table 1:** Drugs used as B-Raf and MEK inhibitors.

Name	IC50	Pathway to Inhibit
SB590885	0.16 nM	B-RAF
AZD6244	14 nM	MEK
	10 nM	ERK ½ Phosphorylation
GDC-0879	63 nM	pERK/B-RAF
Nutlin-3A	90 nM	MDM2/p53
Bortezomib	0.6 nM	ERK/20S Proteasome

**Table 2:** Antibodies used in Simple Western analyses.

Antibody	Dilution	Company
ANXA7	1:50	BD Biosciences, San Jose, CA, USA
B-RAF V600E	1:100	Bio SB Inc, Santa Barbara, CA, USA
ERK	1:100	Cell Signaling Technology, Danvers, MA, USA
P-ERK	1:100	Cell Signaling Technology
Rb	1:100	Cell Signaling Technology
P-Rb	1:100	Cell Signaling Technology
Cyc D1	1:10	Santa Cruz Biotechnology, Dallas, Texas, USA
Cleav. Caspase 3	1:50	Cell Signaling Technology
p21	1:50	BD Biosciences
β-Actin	1:200	Sigma-Aldrich, Burlington, MA, USA
β-Tub	1:50	Upstate Biotech, Syracuse, NY, USA

## Data Availability

Data contained within the article.

## Supplementary Materials

The following supporting information can be downloaded at: https://www.mdpi.com/article/10.3390/ijms252313217/s1.

## Abbreviations

Annexin A7 (ANXA7), cyclin-dependent kinase inhibitor 1 or CDK-interacting protein 1 (p21), v-Raf murine sarcoma viral oncogene homolog B1 (BRAF), Mitogen-Activated Protein Kinase (MAPK), Extracellular signal-Regulated Kinase (ERK), Phosphorylated-ERK (P-ERK), Phosphoinositide 3-kinase (PI3K), Protein Kinase B (Akt), Rat sarcoma viral oncogene homolog (Ras), Rapidly Accelerated Fibrosarcoma (Raf), Mitogen-Activated Protein Kinase (MEK), Extracellular signal-Regulated Kinase (Erk), and Signal Transducer and Activator of Transcription (STAT).
