# Supplementary material for: Role of Annexin 7 (ANXA7) as a Tumor Suppressor and a Regulator of Drug Resistance in Thyroid Cancer
Source: Int J Mol Sci. 2024 Dec 9;25(23):13217. doi: 10.3390/ijms252313217 (PMC11642894; doi:10.3390/ijms252313217)
Supplement: Supplementary file 1 [file ijms-25-13217-s001.zip › ijms-3309394-supplementary.pdf]

Role of Annexin 7 (ANXA7) as a tumor suppressor and a regulator of drug resistance in thyroid cancer

Alakesh Bera[1†#*], Surya Radhakrishnan[1†], Narayanan Puthillathu[1], Madhan Subramanian[1], Nahbuma Gana[1], Eric Russ[1], Harvey Pollard[1], and Meera Srivastava[1]*

Supplementary data:

Table S1. All thyroid cancer cell lines with different mutations. Abbreviation: ms- missense variant; frmv- frameshift variant; blank- Wild type (WT, no mutation). Data analyzed from CCLE data base.

Thyroid Cancer type	Tumor Type	Cell line Name	KRAS	TP53	BRAF	PIK3CA	PTEN
Anaplastic ThCa	Primary	8305C		ms_ (p.R273C)	ms_ (p.V600E)		
Anaplastic ThCa	Primary	8505C		ms_ (p.R248G)	ms_ (p.V600E)		
Anaplastic ThCa	Primary	ASH3		ms_ (p.H179R)			frmv_ (p.E242GfsTer 11)
Poorly Differentiated ThCa	Primary	BCPAP		ms_ (p.D259Y)	ms_ (p.V600E)		
Anaplastic ThCa	Metastatic	BHT101		ms_ (p.I251T)	ms_ (p.V600E)		
Anaplastic ThCa	Primary	CAL62	ms_ (p.G12R)	ms_ (p.A161D)			
Follicular ThCa	Metastatic	FTC133		ms_ (p.R273H)			stop_gained (p.R130Ter)
Follicular ThCa	Metastatic	FTC238		ms_ (p.R273H)			stop_gained (p.R130Ter)
Anaplastic ThCa	Primary	HOTHC		ms_ (p.G244D)			
Anaplastic ThCa	Metastatic	HTCC3		ms_ (p.P152L)	ms_ (p.V600E)		
Papillary ThCa	Metastatic	IHH4			ms_ (p.V600E)		
Papillary ThCa	Metastatic	K5			ms_ (p.V600E)	ms_ (p.E542K)	
Anaplastic ThCa		KMHDASH2		splice_ variant			
Anaplastic ThCa	Primary	MB1		splice_variant			
Follicular ThCa	Primary	ML1		frmv_ (p.P47RfsTer76)			
Follicular ThCa	Metastatic	RO82W1		ms_ (p.P223L)			
Medullary ThCa	Primary	TT					
Follicular ThCa	Primary	TT2609C02		ms_ (p.R273C)			
Papillary ThCa	Primary	K1			ms_ (p.V600E)		
Papillary ThCa	Primary	MDA-T68					

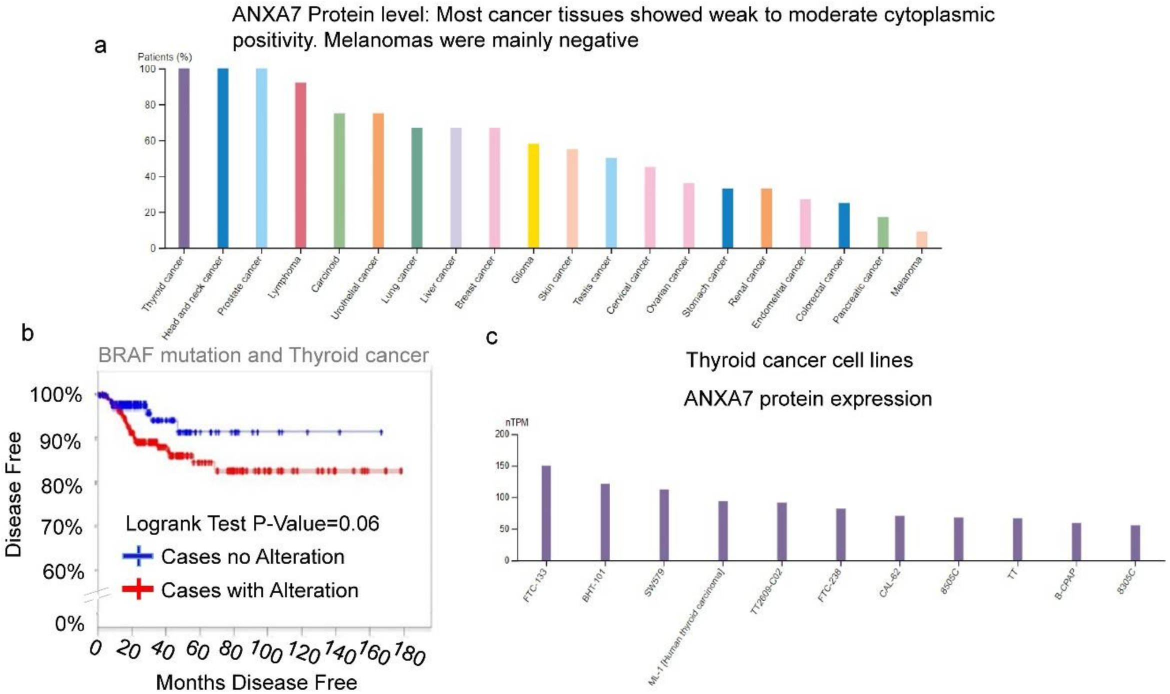

Figure S1. a. ANXA7 expression level in different cancer tissue samples. Data indicated that melanoma tumor tissue has the lowest level of ANXA7. b. Survival curve for thyroid cancer patients in terms of BRAF mutation. c. Protein level of ANXA7 in different thyroid cancer cell lines. Date indicated that 8505C has the lowest level of ANXA7 and FTC-133 has the highest level expression which is matched with our current experimental data at Fig -2.

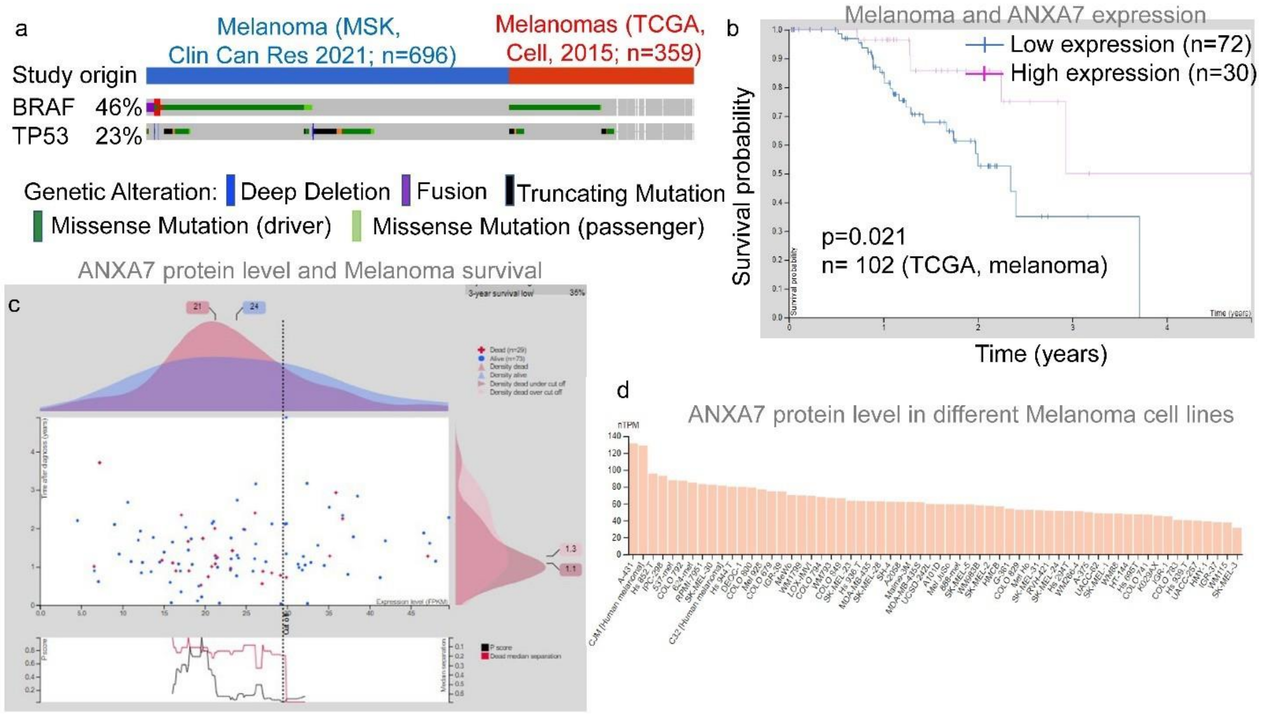

Figure S2. ANXA7 and skin cancer. a. BRAF and p53 mutation status in Melanoma patients tumor samples. b. ANXA7 with Melanoma patients' survival. Survival curve showed pattern with a poor prognosis with low level of ANXA7. c. lower expression of ANXA7 (protein level) showed a poor prognosis (dead patients having average level 21 vs live patients having ANXA7 level 24). C. ANXA7 expression level in different melanoma cancer cell lines. Data analyses were performed from the meat data of cBioportal and human protein atlas and CCLE.
